# Significant Association of Glutathione S-Transferase T1 Null Genotype with Prostate Cancer Risk: A Meta-Analysis of 26,393 Subjects

**DOI:** 10.1371/journal.pone.0053700

**Published:** 2013-01-24

**Authors:** Qing Yang, Jun Du, Xin Yao

**Affiliations:** Department of Genitourinary Oncology, Tianjin Medical University Cancer Hospital, Key Laboratory of Cancer Prevention and Therapy, Tianjin, China; University of Aberdeen, United Kingdom

## Abstract

**Background:**

Recent studies on the association between Glutathione S-transferase T1 (*GSTT1*) polymorphism and risk of prostate cancer showed inconclusive results. To clarify this possible association, we conducted a meta-analysis of published studies.

**Methods:**

Data were collected from the following electronic databases: Pubmed, Embase, and Chinese Biomedical Database (CBM). The odds ratio (OR) and its 95% confidence interval (95%CI) was used to assess the strength of the association. We summarized the data on the association between *GSTT1* null genotype and risk of prostate cancer in the overall population, and performed subgroup analyses by ethnicity, adjusted ORs, and types of controls.

**Results:**

Ultimately, a total of 43 studies with a total of 26,393 subjects (9,934 cases and 16,459 controls) were eligible for meta-analysis. Overall, there was a significant association between *GSTT1* null genotype and increased risk of prostate cancer (OR = 1.14, 95%CI 1.01–1.29, P = 0.034). Meta-analysis of adjusted ORs also showed a significant association between *GSTT1* null genotype and increased risk of prostate cancer (OR = 1.34, 95%CI 1.09–1.64, P = 0.006). Similar results were found in the subgroup analyses by ethnicity and types of controls.

**Conclusion:**

This meta-analysis demonstrates that *GSTT1* null genotype is associated with prostate cancer susceptibility, and *GSTT1* null genotype contributes to increased risk of prostate cancer.

## Introduction

Prostate cancer is a common cause of cancer mortality and one of the most frequently diagnosed malignancies in men [1], [2]. Identifying risk factors for prostate cancer is critically important to develop potential interventions and to expand our understanding of the biology of this disease [2], [3]. Endogenous products and environmental factors could result in the production of reactive oxygen species (ROS) and nitrogen metabolites causing cell injury and genetic instability, and further result in the carcinogenesis in prostate [2]. Glutathione S-transferases (GSTs) play an active role in the detoxification of a variety of endogenous or exogenous carcinogens by glutathione (GSH) conjugation [4], [5]. These enzymes also play a crucial role in protection of DNA from oxidative damage by ROS [4], [5]. In humans, GST super family consists of many cytosolic, mitochondrial, and microsomal proteins, and the cytosolic family has eight distinct classes alpha, kappa, mu, omega, pi, sigma, theta, and zeta [6]. The theta class of GSTs is encoded by the Glutathione S-transferase T1 (*GSTT1*) gene located on the long arm of chromosome 22 (22q11.23), and the homozygous deletion (null genotype) of *GSTT1* gene causes complete absence of GST enzymes activity [7], [8]. In 2009, a meta-analysis on the association between *GSTT1* null genotype and prostate risk was reported. This meta-analysis including 22 studies (3,837 cases and 4,552 controls) concluded that there was no association between *GSTT1* null genotype and prostate risk [9]. Nevertheless, this meta-analysis included relatively small sample size, and many new studies recently have examined the association between *GSTT1* null genotype and prostate risk [10]–[20], but the results remain inconclusive and inconsistent. Hence, to clarify this possible association, we conducted an updated meta-analysis of published studies, which may provide an evidence for the association of *GSTT1* null genotype and prostate risk.

## Materials and Methods

### Identification and Eligibility of Relevant Studies

Data were collected from the following electronic databases: Pubmed, Embase, and and Chinese Biomedical Database (CBM). Relevant publications were identified through a literature search using the following search strategy: (“Glutathione S-transferase T1” or “*GSTT1*” or “*GSTT*”) and (“prostate cancer” or “prostate carcinoma”). Additional literature was collected from cross-references within both original and review articles. No language restrictions were applied. A study was included in the current meta-analysis if: (1) it was published up to May 2012; (2) it was a case-control study; (3) the control subjects are prostate cancer-free regardless of whether they had benign prostate hyperplasia (BPH) or not. We excluded family-based studies of pedigrees with several affected cases per family because the analysis was based on linkage considerations. When a study reported the results on different ethnicities, we treated them as separate studies.

### Data Extraction

Information was carefully extracted from all the eligible publications independently by two of the authors according to the inclusion criteria listed above. Disagreement was resolved by discussion among all authors. Data extracted from the selected studies included author, year of publication, country, ethnicity, definition of cases, characteristics of controls, total numbers of cases and controls, the genotype frequency of *GSTT1* polymorphism, and adjusted odds ratio (OR) and its 95% confidence interval (95%CI). Different descents were categorized as Caucasians, East Asians, Africans, Indians, and Others. If original genotype frequency data were unavailable in relevant articles, a request was sent to the corresponding author for additional data. In deed, only two requests were sent, but no replies were obtained.

### Statistical methods

The strength of the association between *GSTT1* null genotype and prostate cancer risk was assessed by calculating the pooled OR with its 95%CI. The pooled ORs were obtained using either the fixed-effects (Mantel-Haenszel's method) [21] or random-effects (DerSimonian and Laird method) models [22], and the significance of the pooled OR was determined by the Z-test. Heterogeneity assumption was checked by the Chi-square test based Q-statistic [23] and the I^2^ statistic [24]. A significant Q statistic (P<0.10) or I^2^ statistic (I^2^>50%) indicated obvious heterogeneity across studies, and the random effect model was selected to pool the ORs. Otherwise, the fixed effect model was selected to pool the ORs. Subgroup analyses were performed by ethnicity, adjusted ORs, and types of controls. Subgroup analyses were firstly performed by adjusted ORs including subgroup analysis of adjusted ORs and subgroup analysis of unadjusted ORs. Subgroup analyses were then performed ethnicity, and ethnicities were categorized as Caucasians, East Asians, Africans, Indians, and Others. Finally, Subgroup analyses were performed by the types of controls. Publication bias was investigated with the funnel plot. The funnel plot should be asymmetric when there is a publication bias, and the funnel plot asymmetry was further assessed by the method of Egger's linear regression test [25]. Analyses were performed using the software Stata version 11 (StataCorp LP, College Station, TX). A P value less than 0.05 was considered statistically significant, and all the P values were two sided.

## Results

### Characteristics of Eligible Studies

There were 97 papers relevant to the searching words, and 50 papers were excluded (39 overlapping records; 4 were not case-control studies; 3 did not explore *GSTT1* polymorphism; 2 were meta-analysis; 2 were reviews), leaving 47 studies for full publication review [10]–[20], [26]–[61] (Figure S1). Of these, 6 studies were excluded (2 were reviews; 2 were case-only studies; 1 was family-based case-control study; 1 was overlapping study) [56]–[61], leaving 41 studies [10]–[20], [26]–[55] (Figure S1). One study reported the results on two different ethnicities [37] and one study reported the results on two groups [11], and we treated them as separate studies. Finally, a total of 43 independent studies including a total of 26, 393 subjects (9, 934 cases and 16, 459 controls) were used in the current meta-analysis [10]–[20], [26]–[55]. Characteristics of studies eligible for the current meta-analysis were presented in Table 1. 43 independent studies consisted of 21 Caucasians, 6 East Asians, 6 Indins, 2 Africans and 6 mixed populations. Adjusted ORs with corresponding 95%CIs were reported in 13 studies [11]–[13], [26], [28], . There were 7 studies used BPH patients as the controls [10], [12], [17], [18], [29], [35], [51], while only 4 studies used the controls excluding BPH patients [10], [12], [17], [28].

**Table 1:** Characteristics of 43 eligible studies in this meta-analysis.

First author(Year)	Country	Ethnic group	Cases *(GSTT1 Null frequency, %)*	Controls *(GSTT1 Null frequency, %)*	Adjusted variables	Adjusted OR (95%CI)
Choubey VK 2012 (10)	India	Indians	51 prostate cancer cases *(9, 17.6%)*	134 controls without BPH *(17, 12.7%)*; 244 BPH patients as controls *(48, 19.7%)*	None	--
Catsburg C 2012 (Localized) (11)	USA	Mixed	491 localized prostate cancer cases *(80, 16.3%)*	736 controls *(153, 20.8%)*	Age and family history of prostate cancer	1.68 (1.19–2.38)
Catsburg C 2012 (Advanced) (11)	USA	Mixed	909 advanced prostate cancer cases *(162, 17.8%)*	736 controls *(153, 20.8%)*	Age and family history of prostate cancer	1.18 (0.92–1.52)
Hemelrijck MV 2012 (26)	Germany	Caucasians	203 prostate cancer cases *(35, 17.2%)*	360 controls *(64, 17.8%)*	Time of recruitment and family history of prostate cancer	1.08 (0.93–1.25)
Kumar V 2011 (17)	India	Indians	57 prostate cancer cases *(29, 50.9%)*	46 controls without BPH *(22, 47.8%)*; 53 BPH patients as controls *(32, 60.4%)*	None	--
Kwon DD 2011 (16)	Korea	East Asians	166 prostate cancer cases *(85, 51.2%)*	327 controls *(163, 49.8%)*	None	--
Safarinejad MR 2011 (13)	Iran	Caucasians	168 prostate cancer cases *(58, 34.5%)*	336 controls *(70, 20.8%)*	Age, body mass index, occupational status, educational level and smoking status	3.21 (2.52–6.21)
Ashtiani ZO 2011 (18)	Iran	Caucasians	110 prostate cancer cases *(38, 34.5%)*	100 BPH patients as controls *(47, 47.0%)*	None	--
Rodrigues IS 2011 (14)	Brasil	Caucasians	154 prostate cancer cases *(42, 27.3%)*	154 controls *(40, 26.0%)*	None	--
Thakur H 2011 (12)	India	Indians	150 prostate cancer cases *(39, 26.0%)*	172 controls without BPH *(22, 12.8%)*; 150 BPH patients as controls *(18, 12.0%)*	Age, smoking, drinking and non vegetarian diet	2.39 (1.36–4.2)
Norskov MS 2011 (15)	Denmark	Caucasians	128 prostate cancer cases *(26, 20.3%)*	4409 controls *(656, 14.9%)*	None	--
Souiden Y 2010 (20)	Tunisia	Caucasians	110 prostate cancer cases *(30, 27.3%)*	122 controls *(18, 14.8%)*	None	--
Steinbrecher A 2010 (19)	Germany	Caucasians	248 prostate cancer cases *(44, 17.7%)*	492 controls *(77, 15.7%)*	None	--
Lavender NA 2009 (28)	USA	Africans	189 prostate cancer cases *(36, 19.0%)*	584 controls without BPH *(102, 17.5%)*	Age, prostate specific antigen, and west African ancestry	1.15 (0.66–2.02)
Sivonova M 2009 (27)	Slovakia	Caucasians	129 prostate cancer cases *(24, 18.6%)*	228 controls *(45, 19.7%)*	None	--
Lima MM Jr 2008 (29)	Brasil	Caucasians	125 prostate cancer cases *(42, 33.6%)*	100 BPH patients as controls *(22, 22.0%)*	None	--
Davydova NA 2008 (30)	Russia	Caucasians	61 prostate cancer cases *(37, 60.7%)*	100 controls *(43, 43.0%)*	None	--
Mallick S 2007 (31)	Guadeloupe	Mixed	134 prostate cancer cases *(30, 22.4%)*	134 controls *(49, 36.6%)*	None	--
Cunningham JM 2007 (32)	USA	Mixed	499 prostate cancer cases *(185, 37.1%)*	493 controls *(212, 43.0%)*	None	--
Mittal RD 2006 (35)	India	Indians	54 prostate cancer cases *(24, 44.4%)*	105 BPH patients as controls *(30, 28.6%)*	None	--
Lindstrom S 2006 (36)	Sweden	Caucasians	1299 prostate cancer cases *(165, 12.7%)*	728 controls *(107, 14.7%)*	None	--
Yang J 2006 (33)	China	East Asians	163 prostate cancer cases *(89, 54.6%)*	202 controls *(95, 47.0%)*	None	--
Silig Y 2006 (34)	Turkey	Caucasians	152 prostate cancer cases *(34, 22.3%)*	169 controls *(31, 18.3%)*	Age, smoking, and family history of cancer.	1.28 (0.74–2.27)
Agalliu I 2006 (Caucasians) (37)	USA	Caucasians	558 prostate cancer cases *(92, 16.5%)*	522 controls *(88, 16.9%)*	Age, family history of prostate cancer, and PSA testing history.	1.04 (0.73–1.47)
Agalliu I 2006 (Africans) (37)	USA	Africans	31 prostate cancer cases *(7, 20.6%)*	15 controls *(7, 46.7%)*	Age, family history of prostate cancer, and PSA testing history.	0.65 (0.13–3.33)
Nam RK 2005 (40)	Canada	Mixed	996 prostate cancer cases *(189, 19.0%)*	1092 controls *(248, 22.7%)*	Age, ethnic background, family history of prostate cancer, and PSA.	0.81 (0.60–1.0)
Caceres DD 2005 (42)	Chile	Mixed	100 prostate cancer cases *(6, 6.0%)*	129 controls *(14, 10.9%)*	None	--
Srivastava DS 2005 (39)	India	Indians	127 prostate cancer cases *(41, 32.3%)*	144 controls *(29, 20.1%)*	None	--
Wang YL 2005 (38)	China	East Asians	81 prostate cancer cases *(43, 53.1%)*	90 controls *(48, 53.3%)*	None	--
Komiya Y 2005 (41)	Japan	East Asians	186 prostate cancer cases *(112, 60.2%)*	288 controls *(149, 51.7%)*	None	--
Joseph MA 2004 (45)	USA	Caucasians	177 prostate cancer cases *(55, 31.1%)*	265 controls *(61, 23.0%)*	None	--
Mittal RD 2004 (43)	India	Indians	103 prostate cancer cases *(35, 34.0%)*	117 controls *(13, 11.1%)*	None	--
Medeiros R 2004 (44)	Portugal	Caucasians	145 prostate cancer cases *(31, 21.7%)*	184 controls *(44, 23.9%)*	None	--
Nakazato H 2003 (46)	Japan	East Asians	81 prostate cancer cases *(40, 49.4%)*	105 controls *(44, 41.9%)*	None	--
Kidd LC 2003 (47)	Finland	Caucasians	202 prostate cancer cases *(24, 11.9%)*	189 controls *(29, 15.3%)*	None	--
Beer TM 2002 (48)	USA	Caucasians	111 prostate cancer cases *(28, 25.2%)*	146 controls *(33, 22.6%)*	Age	1.0 (0.48–2.08)
Kote-Jarai Z 2001 (50)	UK	Caucasians	273 prostate cancer cases *(67, 24.5%)*	278 controls *(66, 23.7%)*	None	--
Murata M 2001 (49)	Japan	East Asians	115 prostate cancer cases *(68, 59.1%)*	200 controls *(96, 48.0%)*	None	--
Gsur A 2001 (51)	Austria	Caucasians	166 prostate cancer cases *(27, 16.3%)*	166 BPH patients as controls *(33, 19.9%)*	None	--
Kelada SN 2000 (53)	USA	Mixed	256 prostate cancer cases *(60, 23.4%)*	469 controls *(155, 33.0%)*	None	--
Steinhoff C 2000 (52)	Germany	Caucasians	91 prostate cancer cases *(23, 25.3%)*	127 controls *(17, 13.4%)*	None	--
Autrup JL 1999 (55)	Denmark	Caucasians	153 prostate cancer cases *(29, 19.0%)*	288 controls *(44, 15.3%)*	Age at diagnosis	1.31 (0.77–2.19)
Rebbeck TR 1999 (54)	USA	Mixed	232 prostate cancer cases *(186, 80.2%)*	231 controls *(159, 68.8%)*	Age and race	1.83 (1.19–2.80)

### Meta-Analysis

The summary of meta-analysis for *GSTT1* null genotype with prostate cancer risk was shown in Table 2.

**Table 2:** Summary of meta-analysis for *GSTT1* null genotype with prostate cancer risk.

Groups	Studies	Subjects (Cases/Controls)	OR (95%CI)	POR	I2	P heterogeneity
Total studies	43	9934/16012	1.14(1.01–1.29)	0.034	67.2%	<0.001
Subgroup analyses						
Adjusted ORs	13	4343/5387	1.34(1.09–1.64)	0.006	72.8%	<0.001
BPH controls	7	713/918	1.15(0.73–1.80)	0.549	71.1%	0.002
Controls without BPH	4	447/937	1.41(1.06–1.88)	0.020	37.3%	0.189
Caucasians	21	4763/9463	1.17(1.01–1.35)	0.044	50.2%	0.005
East Asians	6	792/1212	1.28(1.07–1.54)	0.007	0.0%	0.727
Africans	2	220/596	0.72(0.23–2.34)	0.571	65.6%	0.088
Indians	6	542/718	2.09(1.60–2.74)	<0.001	27.6%	0.228

(GSTT1, Glutathione S-transferase T1; 95%CI, 95% confidence interval; OR, odds ratio; BPH, benign prostate hyperplasia).

**Figure 1:**
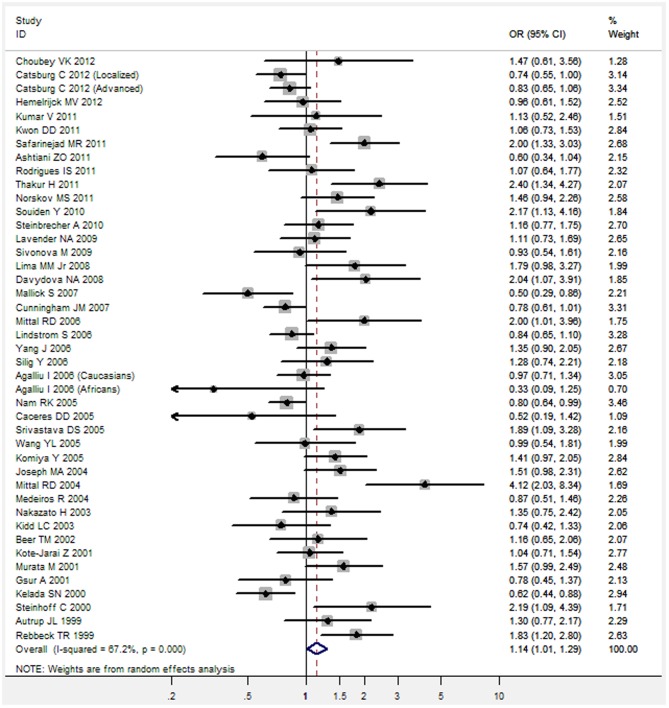
Unadjusted OR with its 95%CI for the association between *GSTT1* null genotype and risk of prostate cancer.

**Figure 2:**
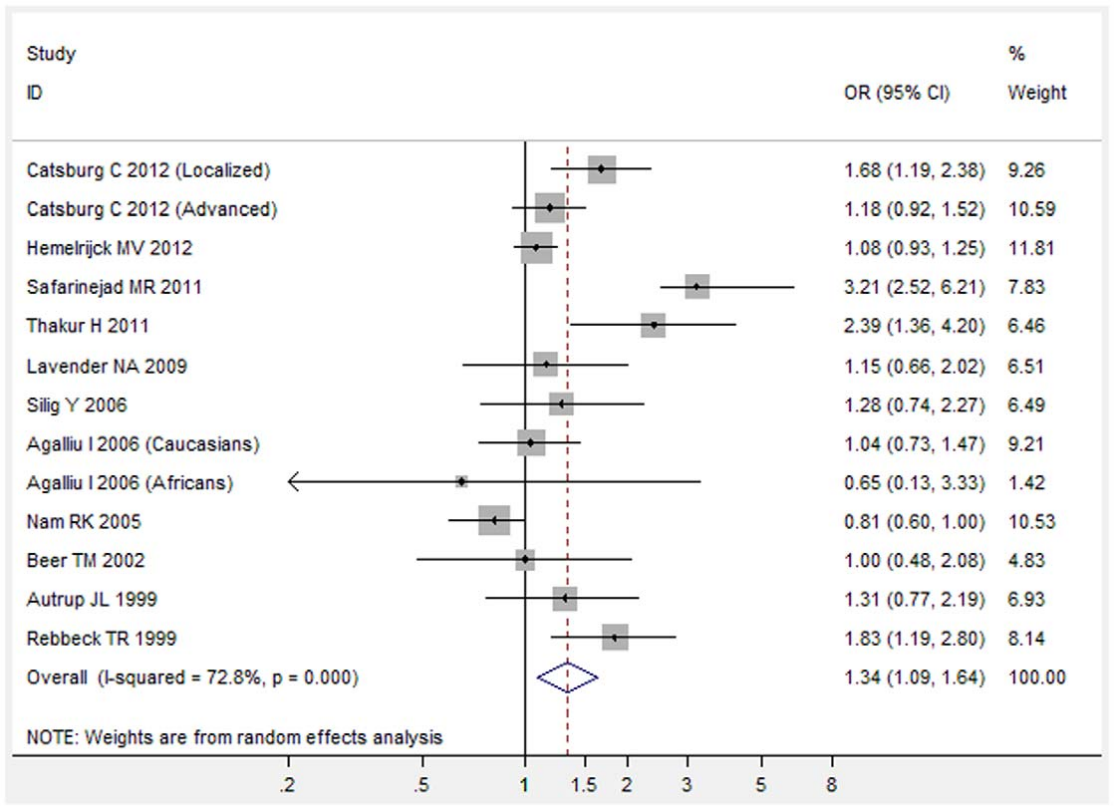
Adjusted OR with its 95%CI for the association between *GSTT1* null genotype and risk of prostate cancer.

Overall, there was a significant association between *GSTT1* null genotype and increased risk of prostate cancer (OR = 1.14, 95%CI 1.01–1.29, P = 0.034) (Figure 1). Meta-analysis of adjusted ORs also showed a significant association between *GSTT1* null genotype and increased risk of prostate cancer (OR = 1.34, 95%CI 1.09–1.64, P = 0.006) (Figure 2).

In the subgroup analyses were firstly performed by ethnicity (Caucasians, East Asians, Africans, and Indians). There was an obvious association between *GSTT1* null genotype and increased risk of prostate cancer in Caucasians (OR = 1.17, 95%CI 1.01–1.35, P = 0.044), East Asians (OR = 1.28, 95%CI 1.07–1.54, P = 0.007), and Indians (OR = 2.09, 95%CI 1.60–2.74, P<0.001), but not in Africans (OR = 0.72, 95%CI 0.23–2.34, P = 0.571).

In the subgroup analysis of BPH controls, there was no obvious association between *GSTT1* null genotype and increased risk of prostate cancer (OR = 1.15, 95%CI 0.73–1.80, P = 0.549). In the subgroup analysis of controls without BPH, there was an obvious association between *GSTT1* null genotype and increased risk of prostate cancer (OR = 1.41, 95%CI 1.06–1.88, P = 0.020).

### Evaluation of Publication Bias

**Figure 3:**
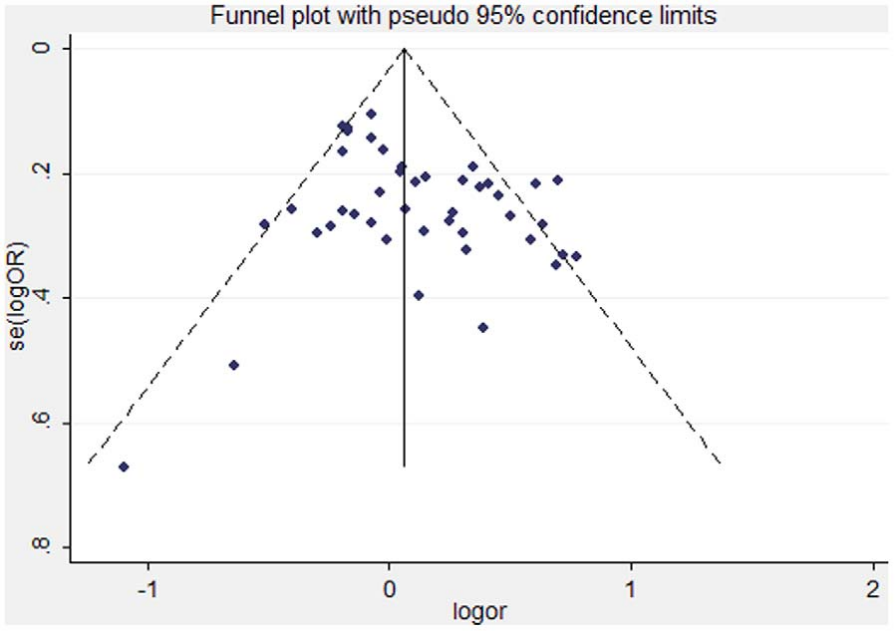
Funnel plot to assess the publication bias of the studies in this meta-analysis.

Both funnel plot and Egger's test were performed to assess the publication bias of the studies. The shape of the funnel plots did not reveal any evidence of obvious asymmetry for any genetic model in the overall and subgroup meta-analysis (Figure 3). Next, Egger's test was used to provide statistical evidence of the funnel plot symmetry. The results still did not suggest any obvious evidence of publication bias for any genetic model (P _Egger's test_ = 0.117). Thus, there was no obvious risk of bias in this meta-analysis.

## Discussion

Genetic susceptibility to cancer has been a research focus and many genetic association meta-analyses have been published to find some possible susceptibility polymorphisms [3], [10]–[20], [26]–[55]. Previous studies assessing the association between *GSTT1* null genotype and prostate cancer risk reported inconclusive and inconsistent findings. Therefore, to get a reliable conclusion for the association of *GSTT1* null genotype and prostate risk, we conducted the present meta-analysis of 43 independent studies including a total of 26, 393 subjects (9, 934 cases and 16, 459 controls). Overall, there was a significant association between *GSTT1* null genotype and increased risk of prostate cancer (Table 2). Meta-analysis of adjusted ORs also showed a significant association between *GSTT1* null genotype and increased risk of prostate cancer (Table 2). Similar association was also found in the subgroup analyses by ethnicity and types of controls (Table 2). Therefore, our meta-analysis demonstrates that *GSTT1* null genotype is associated with prostate cancer susceptibility, and *GSTT1* null genotype contributes to increased risk of prostate cancer.

**Figure 4:**
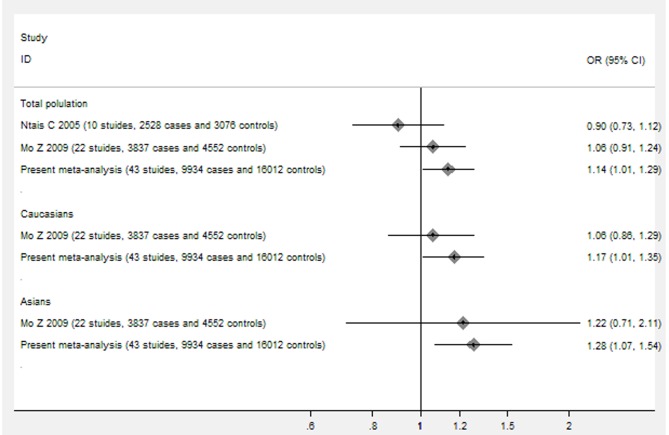
The main differences in the findings between present meta-analysis and previous published meta-analyses.

Previous literature didn't provide a comprehensive assessment on the association between *GSTT1* null genotype and prostate cancer risk, but a trend for potential genetic effects was suggested in early data for the association between *GSTT1* null genotype and prostate cancer risk. Postulated genetic associations for prostate cancer need to be carefully validated, because early and small genetic association studies may come up with spurious findings. Two previous meta-analyses were published to assess the association between *GSTT1* null genotype and prostate cancer risk, but both failed to find a significant association [9], [62] (Figure 4). Compared with those two meta-analyses, our meta-analysis provides several new findings. Our meta-analysis includes much larger participants and more new studies (43 studies, 9, 934 cases and 16, 459 controls) and is the largest meta-analysis of the association between *GSTT1* null genotype and prostate cancer risk. The present meta-analysis has much greater power to detect the real association, and draw a more precise and reliable conclusion. The pooled results in our meta-analysis suggests a significant association between *GSTT1* null genotype and increased risk of prostate cancer, which provides a comprehensive evidence and reliable conclusion for the association above (Figure 4).

In our meta-analysis, the cases and controls have been recruited through different sources. The control subjects in our meta-analysis are defined as cancer-free, and the BPH patients are also enrolled in many included studies in the meta-analysis. Though there is no obvious association between BPH and prostate cancer, there is also a significant association between *GSTT1* polymorphism and BPH and the *GSTT1* null genotype frequency is higher in the BPH patients than that in the healthy controls [10]. Our meta-analysis suggest there is no obvious association between *GSTT1* null genotype and prostate cancer risk in the subgroup analysis of studies with BPH controls, but there is an obvious association between *GSTT1* null genotype and increased risk of prostate cancer in the subgroup analysis of studies with non-BPH controls (Table 2), which indicates this discrepancy in the *GSTT1* null genotype frequency between BPH patients and healthy controls may affect the association between *GSTT1* null genotype and risk of prostate cancer. Since there is also an obvious association between *GSTT1* null genotype and increased risk of BPH, the frequency of *GSTT1* null genotype is much higher in the BPH patients than that in the healthy controls [10]. When one case-control study selects the BPH patients as the controls to assess the association between *GSTT1* null genotype and prostate cancer risk, the higher frequency of *GSTT1* null genotype in the BPH patients may become a major confounding factor and could bias the real estimation of the association between *GSTT1* null genotype and prostate cancer risk [10].

GSTs are the most important family of phase II isoenzymes which are known to detoxify a variety of electrophilic compounds including carcinogens, chemotherapeutic drugs, environmental toxins, and DNA products generated by reactive oxygen species damage to intracellular molecules [4], [6]. GSTs also play a major role in cellular antimutagen and antioxidant defense mechanisms, and these enzymes may regulate pathways that prevent damage from several carcinogens [4], [6]. The null genotype of *GSTT1* gene causes complete absence of GST enzymes activity, decreases the ability of detoxifying electrophilic compounds, and may increase the susceptibility to various cancers [7]. Thus, there is obvious biochemical evidence for the relationship of *GSTT1* null genotype with prostate cancer risk. Besides, *GSTT1* null genotype has also been studied extensively in terms of susceptibility for other malignancies. Previous meta-analyses have yielded significant associations of *GSTT1* null genotype with colorectal cancer [63], breast cancer [64], lung cancer [65] and hepatocellular carcinoma [66], which further suggest *GSTT1* null genotype plays an important role the carcinogenesis and can affect the host susceptibility to common malignancies.

Some limitations of this study should be acknowledged. Firstly, significant between-study heterogeneity was detected in overall analysis, and subgroup analyses in Caucasians and Africans. There are several aspects could explain the significant heterogeneity: the different proportion of BPH patients in the controls, different definition of control group and ethnicity. In addition, it is known that a shorter androgen signaling pathway exist in these individuals from African population, which contributes to prostate cancer risk and may bias the real estimate of the gene-cancer associations in Africans [67]. Therefore, more studies with estimates adjusting for those known risk factors are needed. Secondly, meta-analysis remains retrospective research that is subject to the methodological deficiencies of the included studies. We minimized the likelihood of bias by developing a detailed protocol before initiating the study, by performing a meticulous search for published studies, and by using explicit methods for study selection, data extraction, and data analysis. Thirdly, some misclassification bias is possible. Most studies could not exclude latent prostate cancer cases in the control group. Finally, we could not address gene-gene and gene-environmental interactions. The latter may be important for genes that code proteins with detoxifying function, but would require detailed information on exposures to various potential carcinogens and individual-level data and would be most meaningful only for common exposures that are found to be strong risk factors for the disease.

In conclusion, this study is, to the best our knowledge, the largest meta-analysis of the association between *GSTT1* null genotype and prostate cancer risk. This meta-analysis demonstrates that *GSTT1* null genotype is associated with prostate cancer susceptibility, and *GSTT1* null genotype contributes to increased risk of prostate cancer.

## Supporting Information

Figure S1 **PRISMA 2009 flow diagram in this meta-analysis.**
(TIF)

Checklist S1 **PRISMA Checklist.**
(DOC)
