# Supplementary figures and images for: Significant Association of Glutathione S-Transferase T1 Null Genotype with Prostate Cancer Risk: A Meta-Analysis of 26,393 Subjects
Source: PLoS One. 2013 Jan 24;8(1):e53700. doi: 10.1371/journal.pone.0053700 (PMC3554715; doi:10.1371/journal.pone.0053700)

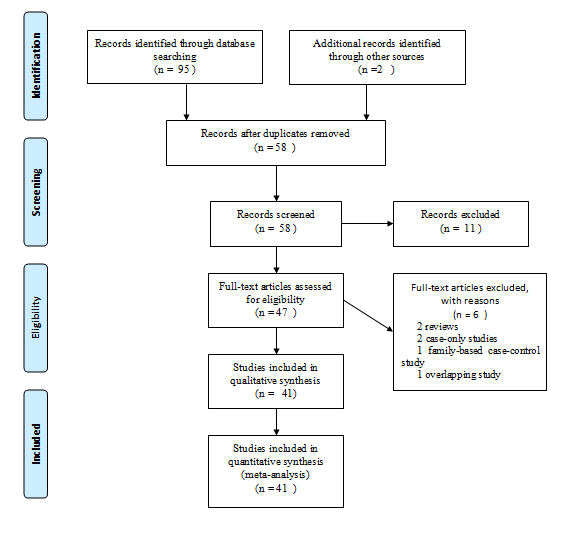

Supplement: Figure S1 — PRISMA 2009 flow diagram in this meta-analysis. (TIF) [file pone.0053700.s001.tif]
